# Thrombotic vs. Bleeding Events of Interruption of Dual Antiplatelet Therapy within 12 Months among Patients with Stent-Driven High Ischemic Risk Definition following PCI

**DOI:** 10.1155/2022/3895205

**Published:** 2022-01-13

**Authors:** Hao-Yu Wang, Bo Xu, Chen-Xi Song, Chang-Dong Guan, Li-Hua Xie, Yan-Yan Zhao, Zhong-Xing Cai, Sheng Yuan, Ke-Fei Dou

**Affiliations:** ^1^Department of Cardiology, Cardiometabolic Medicine Center, Coronary Heart Disease Center, Fuwai Hospital, National Center for Cardiovascular Diseases, State Key Laboratory of Cardiovascular Disease, Chinese Academy of Medical Sciences and Peking Union Medical College, Beijing, China; ^2^Catheterization Laboratories, Fuwai Hospital, National Center for Cardiovascular Diseases, Chinese Academy of Medical Sciences and Peking Union Medical College, Beijing, China; ^3^Medical Research and Biometrics Center, Fuwai Hospital, National Center for Cardiovascular Diseases, Chinese Academy of Medical Sciences, Peking Union Medical College, Beijing, China

## Abstract

**Background:**

There is a paucity of real-world data regarding the clinical impact of dual antiplatelet therapy (DAPT) interruption (temporary or permanent) among patients at high ischemic risk. The aim of this study was to assess the risk of cardiovascular events after interruption of DAPT in high-risk PCI population.

**Methods:**

This study used data from the Fuwai PCI registry, a large, prospective cohort of consecutive patients who underwent PCI. We assessed 3,931 patients with at least 1 high ischemic risk criteria of stent-related recurrent ischemic events proposed in the 2017 ESC guidelines for focused update on DAPT who were free of major cardiac events in the first 12 months. The primary ischemic endpoint was 30-month major adverse cardiac and cerebrovascular events, and the key safety endpoints were BARC class 2, 3, or 5 bleeding and net adverse clinical events.

**Results:**

DAPT interruption within 12 months occurred in 1,122 patients (28.5%), most of which were due to bleeding events or patients' noncompliance to treatment. A multivariate Cox regression model, propensity score (PS) matching, and inverse probability of treatment weighting (IPTW) based on the propensity score demonstrated that DAPT interruption significantly increased the risk of primary ischemic endpoint compared with prolonged DAPT (3.9% vs. 2.2%; Cox-adjusted hazard ratio (HR): 1.840; 95% confidence interval (CI): 1.247 to 2.716; PS matching-HR: 2.049 [1.236–3.399]; IPTW-adjusted HR: 1.843 [1.250–2.717]). This difference was driven mainly by all-cause death (1.8% vs. 0.7%) and MI (1.3% vs. 0.5%). Furthermore, the rate of net adverse clinical events (4.9% vs. 3.2%; Cox-adjusted HR: 1.581 [1.128–2.216]; PS matching-HR: 1.639 [1.075–2.499]; IPTW-adjusted HR: 1.554 [1.110–2.177]) was also higher in patients with DAPT interruption (≤12 months), whereas no significant differences between groups were observed in terms of BARC 2, 3, or 5 bleeding. These findings were consistent across various stent-driven high-ischemic risk subsets with respect to the primary ischemic endpoints, with a greater magnitude of harm among patients with diffuse multivessel diabetic coronary artery disease.

**Conclusions:**

In patients undergoing high-risk PCI, interruption of DAPT in the first 12 months occurred infrequently and was associated with a significantly higher adjusted risk of major adverse cardiovascular events and net adverse clinical events. 2017 ESC stent-driven high ischemic risk criteria may help clinicians to discriminate patient selection in the use of long-term DAPT when the ischemic risk certainly overcomes the bleeding one.

## 1. Introduction

Dual antiplatelet therapy (DAPT) of aspirin and a P2Y_12_ inhibitor has been a therapeutic cornerstone after percutaneous coronary intervention (PCI) or acute coronary syndrome (ACS); however, its optimal duration in different clinical scenarios is currently a matter of debate [1,2]. After PCI with drug-eluting stent (DES) implantation, DAPT is generally recommended for 12 months in ACS patients and for 6 months in patients with stable coronary artery disease (CAD) [3]. Although DAPT is continued beyond 12 months after stenting to offer a broader atherothrombotic risk protection, this risk reduction comes at the cost of an increased risk of bleeding [4]. Based on lower rates of late stent thrombosis with newer-generation DES, the risk of thrombotic events is not increased even with 1 to 6 months of DAPT [5]. Many clinical trials (most of which were relatively small and open-label noninferiority trials) have suggested that the benefits of lower risk of bleeding events with abbreviated DAPT followed by aspirin-based single antiplatelet therapy (SAPT) were counterbalanced by higher rates of stent thrombosis [6–9], while an individual participant data meta-analysis showed that P2Y_12_ inhibitor monotherapy after short DAPT was associated with lower major bleeding and similar risks of fatal and ischemic events compared with traditional DAPT [10].

Considering populations in the trials investigating the optimal minimal duration of DAPT followed by aspirin monotherapy mostly constituted of selected patients undergoing elective noncomplex PCI [11], limited and controversial evidence is available on the value of aspirin-based SAPT after shortened DAPT in intermediate-to-high-risk patients. Observational studies have reported increased risk of myocardial infarction (MI) and adverse cardiac outcomes for patients with DAPT interruptions within 6 months after PCI [12–15]. In that respect, the prognostic significance of interruption or any nonadherence to DAPT in the first 12 months in higher-risk routine practice populations remains unclear. Therefore, using prospective data from a contemporary real-world group of patients undergoing PCI, we focused on a subset of patients who satisfied high ischemic risk criteria based on patient-related clinical and angiographic characteristics and PCI-related features (using the 2017 ESC updates for DAPT guidelines) to estimate the incidence of DAPT interruption (temporary or permanent) in the first 12 months after PCI and evaluate the efficacy and safety of DAPT interruption ≤12 months as compared with longer than 12 months of DAPT for these high-risk patients.

## 2. Methods

### 2.1. Study Population

The Fuwai PCI registry database, which involves prospective recruitment of consecutive patients undergoing PCI with DES placement between January 2013 to December 2013 at the Fuwai Hospital (National Center for Cardiovascular Diseases, Beijing, China), was used for the current retrospective analysis. The present analysis included patients with high ischemic risk defined by 2017 ESC DAPT guidelines who were event free at 12 months. We excluded patients who had a major adverse cardiac or cerebrovascular event (the composite of all-cause death, MI, or stroke), repeat revascularization, stent thrombosis, or Bleeding Academic Research Consortium (BARC) type 3 or 5 bleeding at 12-month follow-up. Demographic and clinical characteristics, angiographic and procedural information, and in-hospital and follow-up outcomes were systematically collected and were prospectively entered into the dedicated database. Institutional review board approval was granted for this research by the ethics committee of Fuwai hospital, and written informed consent was obtained for all participants for participation in this prospective registry. This study was conducted in accordance with the Declaration of Helsinki.

### 2.2. Procedures and Follow-Up

The PCI procedure, including device selection and revascularization strategy, and related management followed standard guidelines at the discretion of the treating physician [16,17]. Aspirin 300 mg and a loading dose of a P2Y_12_ inhibitor (clopidogrel 300 or 600 mg or ticagrelor 180 mg) were given before intervention. After PCI, patients were prescribed 100 mg/day aspirin indefinitely and P2Y_12_ inhibitor (clopidogrel 75 mg once daily or ticagrelor 90 mg twice daily) for 12 months. Detailed information on procedures is shown in the supplementary materials. Clinical follow-up was prospectively conducted via office visit or telephone contact at 30 days, 6 months, 12 months, and annually thereafter. At follow-up, data about patients' clinical status, all interventions received, and outcome events were documented by independent research personnel. Information regarding time of DAPT cessation, which drug (aspirin or P2Y_12_ inhibitor) was stopped, and the reason for stopping treatment was collected. Other possible follow-up information was obtained from hospital readmission, outpatient records, the referring physician and relatives, and external medical records from other hospitals, as necessary.

### 2.3. Definitions and Outcomes

Interruptions of DAPT within 12 months were defined as either a temporary interruption of aspirin and/or P2Y12 inhibitor (interruption of at least 1 days) or a permanent discontinuation (>30 days). Permanent DAPT discontinuation was considered if DAPT was never resumed 30 days after discontinuation. Patients were defined as high ischemic risk if they met at least 1 of the following characteristics according to 2017 ESC guidelines for focused update on DAPT in CAD: diabetes with diffuse multivessel CAD, chronic kidney disease, at least 3 lesions treated, at least 3 stents implanted, a total stent length of more than 60 mm, a bifurcation lesion treated with two stents, and treatment of chronic total occlusion [3]. Adapting the criteria to fit the available information, a subset of patients with at least 1 modified ESC high ischemic risk criteria (without information on previous stent thrombosis on antiplatelet therapy and last patent vessel) was defined.

Given that the clinical variables evaluated in the present analysis were collected at a time when the Academic Research Consortium for High Bleeding Risk (ARC-HBR) definition was not yet available, Supplementary Table 1 illustrates the list of major and minor ARC-HBR criteria and their respective definitions adapted to the current study database. Patients were defined as HBR if they met at least 1 major or 2 minor criteria [18]. Conversely, those not meeting any ARC-HBR criterion or patients with only 1 minor criterion were considered non-HBR.

The primary ischemic endpoint was major adverse cardiac and cerebrovascular events, defined as a composite of all-cause death, MI, or stroke. The key secondary endpoint was clinically relevant bleeding defined by BARC 2-, 3-, or 5-type bleedings [19] and net adverse clinical events, defined as a composite of clinically relevant bleeding and major adverse cardiac and cerebrovascular events. Secondary end points included all-cause death, cardiac death, MI, definite or probable ST, and stroke. Cardiac mortality was defined according to Academic Research Consortium (ARC) criteria as any death because of an immediate cardiac cause, deaths related to the procedure, or undetermined cause of death [20]. The diagnosis of MI was based on the Third Universal Definition of MI [21]. Stent thrombosis was defined as definite or probable stent thrombosis based on the ARC classification [20]. Stroke was defined as a focal loss of neurologic function caused by an ischemic or hemorrhagic event, with residual symptoms lasting at least 24 hours or leading to death [22]. Source documents were obtained for any adverse events or any DAPT cessation. All clinical events were adjudicated by an independent clinical event committee, composed of members who did not participate in patient enrollment for this study. All endpoints were evaluated at 30 months. Median follow-up was 877 days (interquartile range: 808 to 944 days).

### 2.4. Statistical Analysis

Categorical data are described as frequency and percentages and continuous data as means with standard deviations. Statistical significance of differences in continuous variables between patient groups was tested with the use of two-sample Student's *t*-test; the chi-square or Fisher exact test was used for categorical variables. Time-to-event data were plotted using the Kaplan–Meier method and compared using the log-rank test. In an attempt to reduce the impact of treatment selection bias inherent to an observational study, three sensitivity analyses were performed to adjust for confounding factors as much as possible. First, a multivariable Cox proportional hazard regression model was used to estimate the independent effect of DAPT duration on clinical outcome. Factors included in multivariable models were based on variables with *P* < 0.10 in the univariate analyses, along with traditional cardiac risk factors. The variables selected appear in the full model shown in Table 1. Second, the propensity score matching (PSM) method was performed. Propensity scores were created by a multivariable logistic regression model with the dependent variable of DAPT duration and a list of covariates as the independent variables. We matched patients with prolonged DAPT (>12-month) to those with discontinued DAPT within 12 months using the 1 : 1 nearest neighbor approach without replacement with a caliper width of 0.2 SD of the logit of the propensity score. Variables included in the PSM models are presented in Supplementary Table 2. Third, we used the inverse probability of treatment weighting (IPTW) Cox proportional hazard regression model to estimate the average treatment effects. In this approach, the weights for patients treated with interruptions (≤12 months) of DAPT were the inverse of (1-propensity score), and the weights for patients treated with DAPT > 12 months were the inverse of propensity score. Balance between the two groups after PSM and IPTW was evaluated by the standardized difference, using a threshold of less than 10% to indicate a balance. Sensitivity analysis was performed with the entire population (*n* = 4,430), including patients who were followed up for <12 months or presented with adverse events within 12 months after PCI in patients who satisfied high ischemic risk criteria. The consistency of effects on primary ischemic endpoint was also explored across the individual components of the ESC high ischemic risk definition, the number of high ischemic risk criteria fulfilled (1, 2, or 3 or more features), and major subgroups. *P* < 0.05 was considered significant for all analyses. Statistical analyses were conducted using SPSS version 24 (SPSS Inc., Chicago, IL, USA) and R version 3.2.0 (R Foundation for Statistical Computing, Vienna, Austria).

## 3. Results

### 3.1. Cohort Characteristics

**Figure 1:**
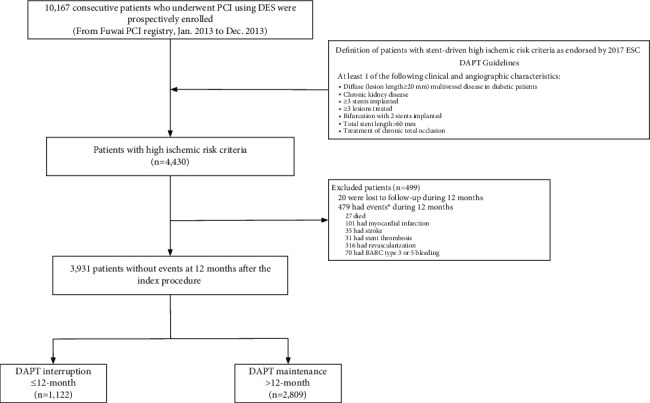
Study flow diagram. BARC = Bleeding Academic Research Consortium; DES = drug-eluting stent; DAPT = dual antiplatelet therapy; ESC = European Society of Cardiology; HIR = high ischemic risk; and PCI = percutaneous coronary intervention. ^*∗*^Subjects may have >1 event.

A total of 10,167 consecutive patients undergoing PCI with DES were eligible for evaluation. Based on the adapted high ischemic risk criteria defined by the 2017 ESC DAPT guidelines, 4,430 patients satisfied at least 1 criterion and were, thus, considered to be at high ischemic risk. Of these, 479 patients with adverse clinical events during the 12-month follow-up after PCI and 20 patients with incomplete follow-up on clinical outcomes within 12 months were excluded. The final cohort consisted of 3,931 patients who were at high ischemic risk and survived the first year after PCI without a major ischemic or bleeding event, of whom 1,122 (28.5%) had an interruption or discontinued DAPT in the first 12 months (Figure 1). Source documents indicated BARC type 1 or 2 bleeding as the most commonly identified reason for the DAPT cessation (42%), followed by nonadherence (31%), need for surgery (12%), other specified reasons (6%), and unknown (9%).

**Figure 2:**
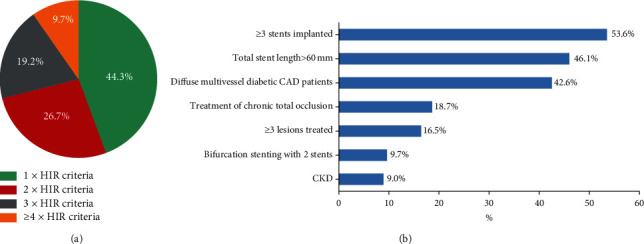
The distribution and prevalence of the ESC stent-driven high ischemic risk criteria components among patients fulfilling high ischemic risk definition. (a) The sum of high ischemic risk criteria satisfied by each patient was used to stratify patients according to the number of times they fulfilled the 2017 ESC DAPT guideline stent-driven high ischemic risk definition. The pie chart shows the distribution of HIR patients with increasing numbers of multiple coexisting criteria (1 × HIR to ≥4 × HIR). (b) Bars indicate the overall prevalence of each high ischemic risk criterion among patients qualified as being at high ischemic risk. HIR = high ischemic risk. Other abbreviations are as in Figure 1.

Mean patient age was 59.1 years; 76.5% were men, 47.2% had diabetes mellitus, and 57.1% presented with acute coronary syndromes (ACS). Nearly 90% of patients had multivessel CAD, and the left main or left anterior descending artery lesion was treated in about 85% of patients. The mean number of ESC high ischemic risk criteria per patient was 2.0; a total of 2,188 of 3,931 patients (55.7%) met 2 or more criteria. The most common high ischemic risk qualifying features were at least 3 stents implanted and diffuse multivessel diabetic CAD patients (Figure 2). Patient-level clinical, angiographic and procedural data in the DAPT interruption ≤12 months and DAPT maintenance > 12 months groups are summarized in Tables 2 and 3. Patients with DAPT interruption ≤12 months more often had ACS as the indication for PCI and had higher rates of ARC for High Bleeding Risk (ARC-HBR) compared with DAPT > 12 months. After PSM and IPTW, the absolute standardized differences for all the baseline patient characteristics between the two groups were less than 0.1 (Supplementary Table 2).

### 3.2. Impact of ESC High Ischemic Risk Criteria on Very Late Clinical Events (12 to 30 Months after PCI)

At 12 to 30 months after stenting, the rate of major adverse cardiac and cerebrovascular events was higher in patients with versus without ESC high ischemic risk criteria (2.7% vs. 1.6%; adjusted HR: 1.533, 95% CI: 1.150–2.044, *P*=0.004), a difference driven by higher rates of all-cause death (1.0% vs. 0.6%), MI (0.8% vs. 0.5%), and stroke (1.5% vs. 0.6%) (Supplementary Table 3). Very late cardiac death was more frequent in patients with versus without ESC high ischemic risk criteria (0.6% vs. 0.3%; HR: 2.405, 95% CI: 1.250–4.626, *P*=0.009). There were no significant differences in the rates of 12- to 30-month clinically relevant bleeding between two groups. After multivariate adjustment, the presence of ESC high ischemic risk remained independently associated with increased 30-month risks for major adverse cardiac and cerebrovascular events, cardiac death, and stroke, with a trend toward an increased risk for MI and stent thrombosis.

### 3.3. Major Adverse Coronary Events and Safety According to the DAPT Treatment Strategy (12 to 30 Months after PCI)

**Figure 3:**
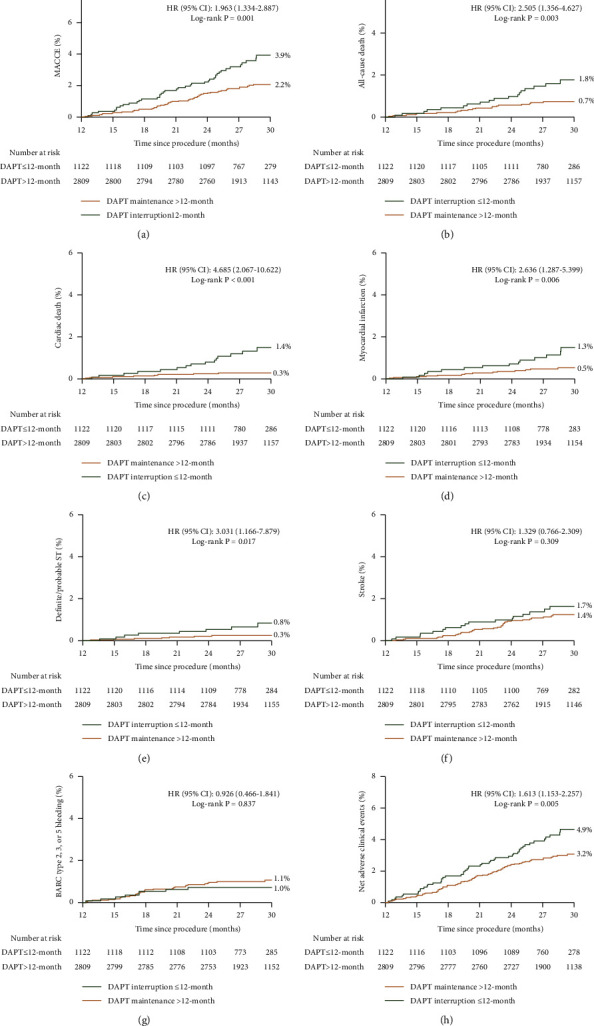
Time-to-event curves in patients with versus without interruption of DAPT within 12 months. (a) Major adverse cardiac and cerebrovascular events; (b) all-cause death; (c) cardiac death; (d) myocardial infarction; (e) stent thrombosis; (f) stroke; (g) BARC type 2, 3, or 5 bleeding; and (h) net adverse clinical events. Numbers at risk are shown below the chart.

Among patients who satisfied high ischemic risk criteria using the 2017 ESC updates for DAPT guidelines, DAPT interruption ≤12 months, compared with extended-term (>12 months) DAPT, had higher crude 30-month rates of major adverse cardiac and cerebrovascular events, all-cause death, cardiac death, MI, and stent thrombosis with similar rates of BARC-defined bleeding type 2, 3, or 5, thereby resulting in an increase in the net adverse clinical events (Figure 3). After multivariable adjustment, cessation of DAPT within 12 months significantly increased the 30-month risk of major adverse cardiac and cerebrovascular events when compared with continued DAPT beyond 12 months (adjusted HR: 1.840, 95% CI: 1.247–2.716; *P*=0.002; Table 1 and Supplementary Table 4), whereas there were no statistically significant differences in BARC 2-, 3-, or 5-type bleedings between the 2 groups (adjusted HR: 0.922, 95% CI: 0.462–1.840; *P*=0.818). Similar trends were observed for cardiac death (adjusted HR: 4.597, 95% CI: 2.011–10.509; *P* < 0.001), MI (adjusted HR: 2.486, 95% CI: 1.209–5.113; *P*=0.013), and stent thrombosis (adjusted HR: 2.979, 95% CI: 1.141–7.783; *P*=0.026). The net adverse clinical events occurred in 91 patients (3.2%) who received DAPT maintenance >12 months and in 55 patients (4.9%) who received DAPT interruption ≤12 months (adjusted HR: 1.581, 95% CI: 1.128–2.216; *P*=0.008).

### 3.4. Sensitivity and Subgroup Analyses

Consistent results were observed when sensitivity analyses using PSM and IPTW were conducted. In the PSM and IPTW propensity score-adjusted cohort, interruption of DAPT within 12 months remained independently associated with increased risks for primary ischemic endpoint (matched HR: 2.049, 95% CI: 1.236–3.399; IPTW-HR: 1.843, 95% CI: 1.250–2.717), cardiac death (matched HR: 5.711, 95% CI: 1.660–19.646; IPTW-HR: 4.786, 95% CI: 2.098–10.914), MI (matched HR: 3.133, 95% CI: 1.137–8.633; IPTW-HR: 2.636, 95% CI: 1.279–5.435), and stent thrombosis (matched HR: 3.204, 95% CI: 0.865–11.869; IPTW-HR: 3.175, 95% CI: 1.227–8.213). There was no significant association between DAPT interruption and BARC type 2, 3, or 5 bleeding (matched HR: 0.796, 95% CI: 0.365–1.740; IPTW-HR: 0.864, 95% CI: 0.430–1.736). A higher risk of net adverse clinical events was identified in subjects with interruptions compared with subjects without (matched HR: 1.639, 95% CI: 1.075–2.499; IPTW-HR: 1.554, 95% CI: 1.110–2.177). Sensitivity analyses conducted for the entire population with stent-driven high ischemic risk definition (*n* = 4,430) exhibited consistent results for the primary and secondary endpoints (Supplementary Tables 5–7), confirming the robustness of the primary analysis. In the entire study population, interruption of DAPT in the first 12 months after PCI was associated with a significantly higher adjusted risk of not only primary ischemic outcome but also the clinically relevant bleeding and net adverse clinical composite outcomes over a 30-month period.

**Figure 4:**
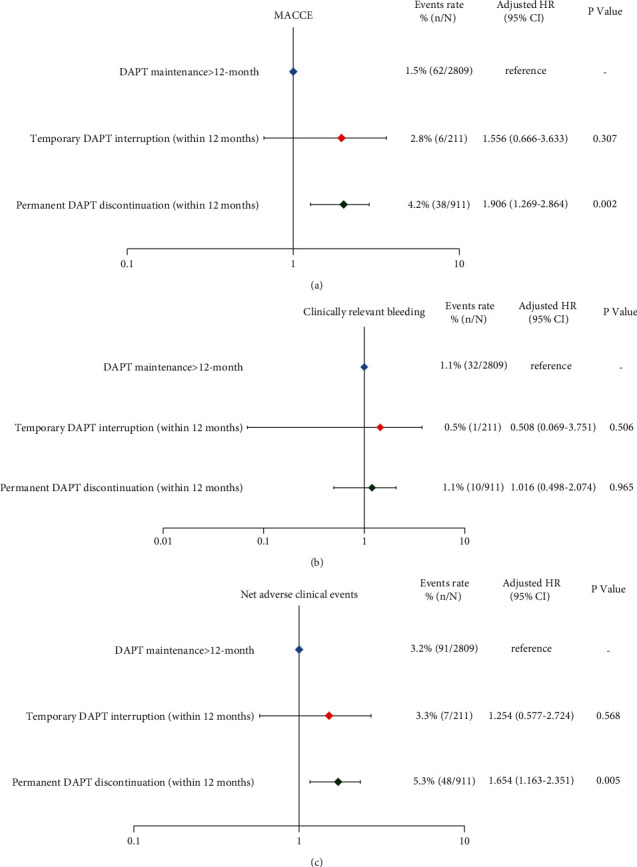
The differential effect of temporary or permanent DAPT interruption within the first 12 months after PCI compared with DAPT maintenance >12 months on primary ischemic and key secondary endpoints. MACCE = major adverse cardiac and cerebrovascular events.

The differential effect of temporary or permanent DAPT interruption within the first 12 months after PCI compared with DAPT maintenance >12 months on primary ischemic and key secondary endpoints is shown in Figure 4 and Supplementary Figure 1. In the patients that were event free after the first year (*n* = 3,931), in contrast to patients who remained on DAPT, the adjusted HR for major adverse cardiac and cerebrovascular events and due to temporary DAPT interruption was 1.556 (95% CI 0.666–3.633; *P*=0.307) and to permanent DAPT discontinuation was 1.906 (1.269–2.864; *P*=0.002) (Figure 4(a)). In supplementary analyses, the entire study population (*n* = 4,430) yielded associations for temporary and permanent DAPT discontinuation that were qualitatively similar in direction and magnitude with our overall findings (Supplementary Figure 1(a)). A similar pattern was observed for net adverse clinical events (Figure 4(c) and Supplementary Figure 1(c)). For clinically relevant bleeding events, patients who had temporary and permanent DAPT discontinuations were not associated with increased risk of major bleeding between 12 and 30 months (Figure 4(b)), while major bleeding throughout the 30-month follow up period was increased only after DAPT interruption on a permanent (>30 days) basis (adjusted HR: 1.706, 95% CI: 1.133–2.596; *P*=0.111) (Supplementary Figure 1(b)).

**Figure 5:**
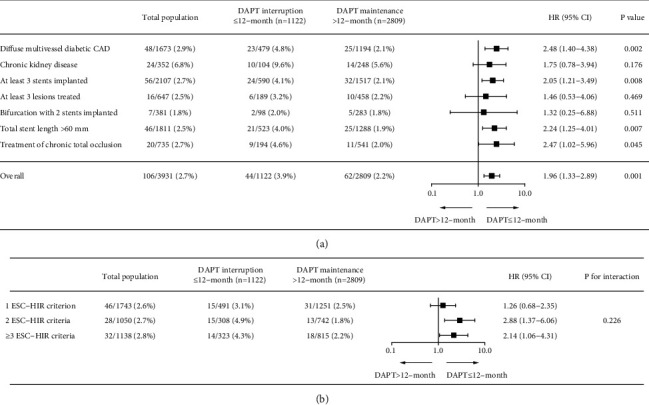
Comparison of long-term risk of primary ischemic endpoint between DAPT interruption ≤12 months and DAPT maintenance > 12 months according to subgroups. The cumulative incidence and hazard ratio with 95% confidence interval of primary efficacy endpoint are presented between DAPT > 12 months and DAPT ≤ 12 months according to the components of the ESC stent-driven high ischemic risk definition (a) and number of ESC stent-driven high ischemic risk criteria fulfilled (b). CAD = coronary artery disease; HIR = high ischemic risk; and other abbreviations are as in Figure 1.

To evaluate the differential effects of DAPT interruption ≤12 months compared with prolonged treatment with DAPT beyond 12 months for various high ischemic risk features according to the 2017 ESC updates for DAPT guidelines, we additionally performed a subgroup analysis according to the components of 2017 ESC high ischemic risk definition (Figure 5(a)). The relationship between DAPT interruption ≤12 months and primary ischemic endpoint was consistent across various clinical or angiographic subsets of high ischemic risk factors. The greatest increased risk in primary ischemic endpoint associated with interruption of DAPT within 12 months was found in patients with diffuse multivessel diabetic CAD (HR: 2.48; 95% CI: 1.40–4.38; *P*=0.002). Relative treatment effects of DAPT interruption ≤12 months were consistent independent of the progressive number of high ischemic risk criteria fulfilled (Figure 5(b)).

We calculated the results of the subgroup analyses comparing the association between DAPT cessation within 12 months and major adverse cardiac and cerebrovascular events for each key subgroup (Table 4 and Supplementary Table 8). In general, the associations were similar in direction and magnitude across key subgroups, and results of formal interaction testing were not significant.

## 4. Discussion

To our knowledge, the present analysis is the first study to date to examine the efficacy and safety of DAPT interruption ≤12 months versus DAPT maintenance >12 months after PCI in real-world patients at stent-driven high ischemic risk criteria undergoing PCI with DES, using data from a contemporary prospective cohort. The principal findings of this study are as follows: (1) interruption (temporary or permanent) with the prescribed 1 year of DAPT (28.5%) was frequent within 12 months of stent implantation, in which the most common reason of DAPT interruption was bleeding or noncompliance; (2) DAPT interruption significantly increased the risk of primary ischemic endpoint (including death, MI, or stroke) up to 30 months; and (3) there was also a modest but statistically significant increase in 30-month net adverse clinical events in those who discontinued DAPT prematurely. Importantly, the main driver of the difference was an increase in all-cause death and MI without resulting in fewer bleeding events. Taken together, patients who stop DAPT prematurely may require more intensive surveillance to prevent long-term adverse events. The current study provided evidence favoring extended-term DAPT therapy for ischemic events, which may be appropriate for certain patients who are at a higher risk of cardiovascular events after PCI and low risk of bleeding, such as those presenting with stent-driven high ischemic risk criteria.

Prescribers of DAPT are confronted with a number of challenges for optimal clinical decision making of DAPT type and duration with the scope of minimizing the risk of ischemic and bleeding events in light of each patient's atherothrombotic and hemorrhagic risk and clinical characteristic and circumstance [1,2]. Although previous studies showed no significant differences in antithrombotic efficacy between short- and long-term DAPT, longer DAPT treatment was associated with an increased risk for bleeding [6–9]. In most studies, patients had a relatively low risk of recurrent ischemia (mostly patients with chronic coronary syndrome or low-risk ACS). However, whether this short DAPT protects sufficiently against ischemic events and adequately reduces bleeding events among patients at high risk of future stent-driven ischemic events is still unclear because of limited statistical power of the individual trials and mixed results [23]. Of note, evidence regarding decisions about the duration of DAPT for patients at high ischemic risk undergoing PCI in real-world clinical practice is scarce. To address this complex issue, we analyzed the risk of major adverse cardiac and cerebrovascular events in subjects who interrupt temporarily or permanently DAPT in the first 12 months after PCI and in patients who met criteria for ESC high ischemic risk and were not at high bleeding risk.

In our cohort, the rate of any interruption of DAPT was 28.5% at 12 months after PCI, which was in line with the post hoc analysis of ADAPT-DES and other registries that assessed the incidence and effect of DAPT cessation on subsequent cardiovascular risk among patients who underwent PCI with DES implantation [24]. A higher incidence of DAPT cessation (30.2%) through 1 year of follow-up has been reported in patients undergoing extensive and more complex PCI in the ADAPT-DES registry [25]. Similarly, any nonadherence to DAPT occurred frequently in the contemporary PCI setting, ranging from 5.1% within 6 months after coronary stents in the DAPT study [12] and 9.6% during the first 6 months after second-generation DES placement in the EDUCATE registry [13] to 23.3% over 1 year of follow-up for patients undergoing PCI in the PARIS registry [15] and 44.0% in patients who discontinued DAPT prematurely (≤12-month) in the Veterans Affairs healthcare system [24].

Our findings extended insights from the ongoing debate regarding the timing and risk of DAPT interruption in patients treated with current-generation DES. Although short-term to midterm (≤6 months) DAPT had similar safety and effectiveness in comparison with 12-month DAPT and better safety than extended-term DAPT, however, these strategies had a higher risk of MI and stent thrombosis than extended-term DAPT, in turn raising concern about broad application of this practice in higher-risk routine population [26]. We enrolled patients at stent-driven high ischemic risk criteria that were more akin to real-world PCI practice patterns and showed higher long-term risks of cardiac mortality and ischemic events after PCI among patients who had DAPT cessation within 12 months, and these risks persisted even in patients who were free of adverse events in the first year. Recently, Sorrentino et al. [27] demonstrated that disruption of DAPT due to bleeding or poor compliance was associated with an increased risk of major adverse cardiac events and MI at 2 years in patients with history of MI, stroke, or peripheral artery disease. It is important to note that the problem of DAPT nonadherence remains inconclusive for the first 6 months after DES placement. Data from previous studies also showed that discontinuation of DAPT within 6 months of stenting was associated with significantly higher risk of thrombotic complications, including death, MI, and stent thrombosis [12,13,28]. Our registry amplified these findings by identifying an independent association of major adverse cardiac events with DAPT interruption ≤12 months, reflecting that physicians appropriately continue DAPT beyond 12 months in high-ischemic risk patients, thereby accounting for lower ischemic events after adopting an extended DAPT strategy.

Previous studies attempted to evaluate the effect of different modes of DAPT cessation on cardiac events after PCI [15,29]. The Xience V coronary stent system trials suggested that the rate of definite and probable ST after cobalt chromium everolimus-eluting stents in patients interrupting DAPT at any point was similar to that of patients who never interrupted DAPT during the 2-year follow-up period, whereas permanent DAPT discontinuation before 3 months was strongly associated with ST in a large, pooled sample of real-world patients [29]. Additionally, Mehran et al. [15] detected no significant increase in thrombotic events in patients who had temporary DAPT interruption lasting up to 14 days. The present study confirms and extends these findings by demonstrating that DAPT interruption on a permanent (>30 days) basis within 1 year after DES implantation was associated with higher 30-month rate of major adverse cardiac and cerebrovascular events in patients at increased ischemic risk, while temporary interruption of DAPT did not influence the rate of ischemic events at 30 months. These findings were consistent for patients without experiencing major adverse events of the first 12 months after PCI and for the total study cohort.

A careful assessment of both bleeding and ischemic risks of the individual patient represents the foundation towards personalized medicine in the field of antiplatelet therapy. In the field of managing bleeding vs. ischemic outcomes, we need to optimize therapies for those at HBR after PCI as a post-PCI bleeding event confers an adverse prognosis similar to post-PCI myocardial infarction [30]. Recently, the ARC-HBR criteria were established to standardize the definition of HBR and promote consistency across trials evaluating this vulnerable subset of patients [31]. A number of studies have reported on the predictive value of the ARC-HBR definition in identifying patients at increased risk not only for bleeding but also for thrombotic events [18,32–34], as well as validated the clinical usefulness of the ARC-HBR criteria in relation to clinical presentation and sex [35,36]. Given that bleeding risk was a major reason for discontinuation of DAPT, we also assessed the effects of DAPT interruption ≤12 months compared with DAPT maintenance >12 months in a contemporary PCI population at high ischemic risk and HBR (Table 4). We found that there was no significant difference in the primary ischemic endpoint between treatment arms, irrespective of ARC-HBR status. In aggregate, disruption of DAPT was associated with an increased risk of major adverse cardiac events in high-ischemic risk patients irrespective of the underlying bleeding risk.

In this contemporary cohort of high-risk patients undergoing PCI, the relation between DAPT interruption within 12 months and thrombotic risk is multifactorial and likely both associative and causative in nature. First, in the current analysis, the cumulative incidence of DAPT interruption within 12 months after PCI was higher in ARC-HBR patients, and the higher propensity of HBR patients in developing hemorrhagic complications lead to permanent cessation of DAPT. This finding further emphasizes that DAPT interruption per se may represent a marker of patient risk. Interruption of DAPT in the setting of major bleeding will inevitably result in permanent and abrupt cessation of antithrombotic therapies, need for blood transfusions, invasive procedures to manage bleeding, and their clinical consequences. Second, more and longer stents implanted may increase the likelihood of stent size mismatch, stent underexpansion, malapposition, and overlapping, all of which may act as true mediators of delayed endothelization and enhancing the stent-related thrombotic risk [37]. In the current study, up to one-half of patients received at least 3 stents implanted coexisting with a higher proportion of total stent length >60 mm, a fact that suggests that encouraged us to prevent premature discontinuation of DAPT and treat them appropriately (e.g., extended DAPT periods). Third, given that patients with multivessel CAD represent an advanced state of atherosclerosis and often leads to incomplete revascularization with a subsequently increased risk of recurrent atherothrombotic coronary events and mortality [38,39], nearly 90% of the population in our cohort comprised of multivessel CAD and, thus, constitute a high-risk patient group that longer duration of DAPT may be appropriate to mitigate ischemic risks both within and outside of the stented segments. Finally, this large PCI cohort reflecting a real-world setting showed that procedural complexity as assessed by ESC stent-driven high ischemic risk definition adequately captures a patient's cardiovascular and noncardiovascular comorbidities with a greater probability of natural plaque progression followed by future atherothrombotic events, particularly in nontarget lesion events. As reported previously, ischemic events may arise from either stented segments or progressive disease elsewhere in the coronary vasculature >1 year after PCI. Late stent-related events were related to patient age, diabetes, and coronary lesion complexity [40]. In this regard, the fatal impact of premature discontinuation of DAPT within 1 year in our dataset might be driven by increasing recurrent ischemic events arising from either from the stented target lesion and nonrevascularized atherosclerotic plaques. Of note in the SWEDEHEART registry, the risk of recurrent MI not originating from a previously stented lesion was twice as high as the risk of lesions originating from a previously stented lesion, emphasizing the importance of preventing atherothrombotic events from nontreated lesions long term and overall coronary disease progression after an initial MI [41]. Collectively, our findings provided additional insights to the expanding evidence base for utilizing 2017 ESC stent-driven high ischemic risk criteria to guide clinical practice to identify patients who might benefit from prolonging antithrombotic treatment duration.

### 4.1. Limitations

Certain limitations were present with the analyses presented within this study. First, our data were derived from a large volume single-center PCI registry and may suffer from limited generalizability. Second, although the data were collected prospectively, as for any retrospective study, our findings should be considered hypothesis generating, and future trials are needed to confirm our findings. We performed additional sensitivity analyses, but there is a possibility of unmeasured confounding factors that may lead to increased risk of ischemic events associated with subjects who had an interruption or discontinued DAPT. Third, the decision to discontinue or remain on DAPT after 12 months was made at the discretion of the patient's physician (and possibly influenced by the patient); hence, selection bias was inevitable in this specific substudy cohort that might have affected event rates. Fourth, ticagrelor (0.4%) only became available late during the study recruitment, and prasugrel was unavailable in China; as a result, most of patients received clopidogrel as a P2Y_12_ inhibitor for DAPT. However, owing to the differential propensity for bleeding events with response to antiplatelet therapy, East Asian populations undergoing PCI treated with potent P2Y_12_ inhibitors did not have a lower ischemic outcome, but had higher incidence of clinically significant bleeding compared with clopidogrel [42,43]. In this scenario, our patients were less prone to bleeding and were more likely to show a net clinical outcome. Furthermore, although CYP2C19 genotyping might be used as an optional tool for guiding antiplatelet therapy, it was not available for this study. Finally, our study was performed in a Chinese population. Compared with Western population, East Asian population has a higher prevalence of the CYP2C19 loss-of-function genotype, which is associated with a higher level of platelet reactivity during clopidogrel treatment. Thrombogenicity, pharmacogenetics, and susceptibility for bleeding complication on P2Y12 inhibitors could be different between Asian and Western population. Thus, we should be cautious about extrapolating these study results outside China.

## 5. Conclusions

In this large-scale PCI cohort of patients with stent-driven high ischemic risk definition, interruption of DAPT (temporary or permanent) predominantly due to poor compliance or bleeding complications within 12 months was associated with significantly higher risk for cardiovascular ischemic recurrences at 30 months compared with prolonged-term (>12 months) DAPT. Patients who had DAPT interruption may benefit from more intensive surveillance to prevent long-term cardiovascular events. Our findings may inform future considerations for utilizing ESC stent-driven high ischemic risk criteria to help clinicians adopting a prolonged DAPT course to pursue a best benefit-risk ratio in an individual patient.

## Figures and Tables

**Table 1:** 30-month ischemic and bleeding outcomes by DAPT interruption status.

	DAPT interruption ≤12 months (*n* = 1122)	DAPT maintenance >12 months (*n* = 2809)	Multivariable adjusted^*∗*^		Propensity score matching		IPTW adjusted		Unadjusted	
			HR (95% CI)	*P* value	HR (95% CI)	*P* value	HR (95% CI)	*P* value	HR (95% CI)	*P* value
Major adverse cardiac and cerebrovascular events^a^	44 (3.9)	62 (2.2)	1.840 (1.247–2.716)	0.002	2.049 (1.236–3.399)	0.005	1.843 (1.250–2.717)	0.002	1.963 (1.334–2.887)	0.001
All-cause death	20 (1.8)	21 (0.7)	2.510 (1.353–4.653)	0.003	2.679 (1.177–6.096)	0.019	2.513 (1.357–4.653)	0.003	2.505 (1.356–4.627)	0.003
Cardiac death	16 (1.4)	9 (0.3)	4.597 (2.011–10.509)	<0.001	5.711 (1.660–19.646)	0.006	4.786 (2.098–10.914)	<0.001	4.685 (2.067–10.622)	<0.001
Myocardial infarction	15 (1.3)	15 (0.5)	2.486 (1.209–5.113)	0.013	3.133 (1.137–8.633)	0.027	2.636 (1.279–5.435)	0.009	2.636 (1.287–5.399)	0.008
Stent thrombosis (definite/probable)	9 (0.8)	8 (0.3)	2.979 (1.141–7.783)	0.026	3.204 (0.865–11.869)	0.081	3.175 (1.227–8.213)	0.017	3.031 (1.166–7.879)	0.023
Stroke	19 (1.7)	38 (1.4)	1.275 (0.730–2.224)	0.393	1.349 (0.684–2.662)	0.387	1.317 (0.761–2.280)	0.326	1.329 (0.766–2.309)	0.312
Clinically relevant bleeding^b^	11 (1.0)	32 (1.1)	0.922 (0.462–1.840)	0.818	0.796 (0.365–1.740)	0.568	0.864 (0.430–1.736)	0.681	0.926 (0.466–1.841)	0.827
Net adverse clinical events^c^	55 (4.9)	91 (3.2)	1.581 (1.128–2.216)	0.008	1.639 (1.075–2.499)	0.022	1.554 (1.110–2.177)	0.010	1.613 (1.153–2.257)	0.005

Values are number of events (Kaplan–Meier estimated event rates), unless otherwise indicated. ^*∗*^Adjusted variables included age, sex, body mass index, current smoking, hypertension, diabetes mellitus, left ventricular ejection fraction, peripheral artery disease, prior coronary artery bypass grafting, prior myocardial infarction, prior PCI, prior major bleeding, acute coronary syndrome presentation, transradial approach, use of intravascular ultrasound, drug-eluting stent type, and total stent length. ^a^Major adverse cardiac and cerebrovascular events included the composite of all-cause mortality, myocardial infarction, or stroke; ^b^clinically relevant bleeding was defined as BARC type 2, 3, or 5 bleeding; ^c^net adverse clinical events included the composite of all-cause mortality, myocardial infarction, stroke, or clinically relevant bleeding. CI, confidence interval; CABG, coronary artery bypass grafting; DAPT, dual antiplatelet therapy; HR, hazard ratio; PCI, percutaneous coronary intervention.

**Table 2:** Baseline characteristics.

	Interruption of DAPT within 12 months after PCI		*P* value
	No (*n* = 2809)	Yes (*n* = 1122)	
Age, years	59.05 ± 10.00	59.33 ± 10.48	0.445
Male	2155 (76.7)	854 (76.1)	0.687
Hyperlipidemia	1954 (69.6)	751 (66.9)	0.108
Hypertension	1926 (68.6)	764 (68.1)	0.773
Diabetes mellitus	1309 (46.6)	545 (48.6)	0.263
Chronic kidney disease^*∗*^	248 (8.8)	104 (9.3)	0.662
Current smoker	1601 (57.0)	627 (55.9)	0.525
Heart failure	69 (2.5)	22 (2.0)	0.351
Peripheral artery disease	89 (3.2)	38 (3.4)	0.727
History of myocardial infarction	607 (21.6)	240 (21.4)	0.880
Prior PCI	630 (22.4)	256 (22.8)	0.792
Prior CABG	139 (4.9)	53 (4.7)	0.768
History of stroke	331 (11.8)	152 (13.5)	0.128
History of major bleeding^†^	17 (0.6)	12 (1.1)	0.124
Body mass index, kg/m^2^	26.10 ± 3.14	25.97 ± 3.28	0.279
LVEF, %	62.42 ± 7.63	62.42 ± 7.46	0.991
Clinical presentation			0.010
Stable coronary artery disease	1241 (44.2)	445 (39.7)	
Acute coronary syndrome	1568 (55.8)	677 (60.3)	
UA/NSTEMI	1234 (43.9)	527 (46.9)	0.084
STEMI	334 (11.9)	150 (13.4)	0.203
White blood cell count, 10^9^/L	6.80 ± 1.64	6.84 ± 1.63	0.482
Hemoglobin, g/dL	14.25 ± 1.58	14.18 ± 1.56	0.220
Platelet count, 10^9^/L	204.08 ± 53.91	205.22 ± 53.21	0.550
ARC-HBR	573 (20.4)	264 (23.5)	0.032
Discharge medication			
Aspirin	2778 (98.9)	1112 (99.1)	0.554
Clopidogrel	2766 (98.5)	1110 (98.9)	0.266
Ticagrelor	9 (0.3)	8 (0.7)	0.107
*β*-blocker	2584 (92.0)	1033 (92.1)	0.935
ACEI/ARB	1731 (61.6)	694 (61.9)	0.893
CCB	1408 (50.1)	550 (49.0)	0.531
Statin	2701 (96.2)	1068 (95.2)	0.168

Values are mean ± SD for continuous variables and *n* (%) for categorical variables. ACS indicates acute coronary syndrome; ACEI, angiotensin-converting enzyme inhibitors; ARB, angiotensin II receptor antagonists; ARC-HBR, Academic Research Consortium-High Bleeding Risk; CCB, calcium channel blockers; CABG, coronary artery bypass grafting; DAPT, dual antiplatelet therapy; LVEF, left ventricular ejection fraction; NSTEMI, non-ST-segment elevation myocardial infarction; PCI, percutaneous coronary intervention; STEMI, ST-segment elevation myocardial infarction; and UA, unstable angina. ^*∗*^Chronic kidney disease was defined as an estimated glomerular filtration rate of less than 60 mL/min/1.73 m^2^ of body surface area. ^†^Spontaneous (nonintracranial) bleeding requiring hospitalization or transfusion.

**Table 3:** Lesion and procedural characteristics.

	Interruption of DAPT within 12 months after PCI		*P* value
	No (*n* = 2809)	Yes (*n* = 1122)	
Lesion characteristics			
Multivessel CAD	2525 (89.9)	998 (88.9)	0.382
Location of the lesion treated			
LM	140 (5.0)	50 (4.5)	0.486
LAD	2386 (84.9)	964 (85.9)	0.436
LCx	835 (29.7)	348 (31.0)	0.426
RCA	959 (34.1)	371 (33.1)	0.520
Bypass graft	7 (0.2)	3 (0.3)	0.919
Target lesion morphology			
Heavy calcified lesion	143 (5.1)	55 (4.9)	0.807
In-stent restenosis lesion	128 (4.6)	49 (4.4)	0.796
Bifurcation lesion	569 (20.3)	221 (19.7)	0.693
Bifurcation with two stents implanted	283 (10.1)	98 (8.7)	0.200
Thrombotic lesion	116 (4.1)	52 (4.6)	0.480
Chronic total occlusion	541 (19.3)	194 (17.3)	0.153
Type B2 or C lesion	2510 (89.4)	1020 (90.9)	0.146
SYNTAX score	14.67 ± 8.46	14.51 ± 8.26	0.629
Total lesion length, mm	57.22 ± 30.35	55.76 ± 27.88	0.163
Procedural characteristics			
Number of vessels treated	1.49 ± 0.60	1.50 ± 0.59	0.566
Number of lesions treated	1.75 ± 0.81	1.75 ± 0.81	1.000
1	1241 (44.1)	501 (44.7)	0.787
2	1111 (39.6)	432 (38.5)	0.543
≥3	458 (16.3)	189 (16.8)	0.680
Number of stents implanted	2.66 ± 1.16	2.59 ± 1.13	0.105
1	429 (15.3)	189 (16.8)	0.221
2	863 (30.7)	343 (30.6)	0.926
≥3	1517 (54.0)	590 (52.6)	0.420
Total stent length, mm	61.00 ± 29.34	59.99 ± 27.90	0.327
Total stent length > 60 mm	1280 (45.9)	523 (46.6)	0.666
Mean stent diameter, mm	2.92 ± 0.52	2.91 ± 0.54	0.640
Vascular access site			0.459
Radial approach	2538 (90.4)	1005 (89.6)	
Femoral approach	271 (9.6)	117 (10.4)	
Use of intravascular ultrasound	214 (7.6)	91 (8.1)	0.602
Use of glycoprotein IIb/IIIa inhibitors	520 (18.5)	223 (19.9)	0.324
Drug-eluting stent type			0.949
First-generation DES	286 (10.2)	115 (10.2)	
Second-generation DES	2523 (89.8)	1007 (89.8)	

Values are mean ± SD for continuous variables and *n* (%) for categorical variables. CAD indicates coronary artery disease; DES, drug-eluting stent; LM, left main coronary artery; LAD, left anterior descending coronary artery; LCx, left circumflex coronary artery; RCA, right coronary artery; and SYNTAX, Synergy between PCI with Taxus and Cardiac Surgery.

**Table 4:** Primary ischemic endpoint in selected subgroups.

	DAPT interruption ≤12 months (*n* = 1122)	DAPT maintenance >12 months (*n* = 2809)	HR (95% CI)	*P* for interaction
Age				0.570
<65 years	21/765 (2.7%)	34/1983 (1.7%)	1.688 (0.979–2.912)	
≥65 years	24/358 (6.7%)	27/825 (3.3%)	2.168 (1.250–3.762)	
Sex				0.470
Female	12/268 (4.5%)	12/654 (1.8%)	2.454 (1.102–5.466)	
Male	33/855 (3.9%)	49/2154 (2.3%)	1.829 (1.175–2.849)	
Diabetes mellitus				0.350
No	20/578 (3.5%)	33/1499 (2.2%)	1.596 (0.914–2.786)	
Yes	25/545 (4.6%)	28/1309 (2.1%)	2.346 (1.365–4.034)	
Chronic kidney disease				0.834
No	35/1019 (3.4%)	47/2560 (1.8%)	2.026 (1.306–3.142)	
Yes	10/104 (9.6%)	14/248 (5.6%)	1.751 (0.778–3.943)	
Smoking				0.182
No	24/495 (4.8%)	24/1208 (2.0%)	2.577 (1.462–4.542)	
Yes	21/628 (3.3%)	37/1600 (2.3%)	1.545 (0.903–2.644)	
Acute coronary syndrome				0.310
No	18/446 (4.0%)	22/1240 (1.8%)	2.479 (1.326–4.635)	
Yes	27/677 (4.0%)	39/1568 (2.5%)	1.632 (0.996–2.673)	
Previous MI				0.440
No	31/882 (4.0%)	46/2202 (2.1%)	1.767 (1.120–2.788)	
Yes	14/241 (5.8%)	15/606 (2.5%)	2.531 (1.212–5.287)	
Multivessel disease				0.284
No	6/125 (4.8%)	4/283 (1.4%)	3.683 (1.037–13.083)	
Yes	39/998 (3.9%)	57/2525 (2.3%)	1.813 (1.206–2.728)	
Generation of DES				0.889
First-generation DES	4/115 (3.5%)	6/286 (2.1%)	2.030 (0.562–7.340)	
Second-generation DES	41/1008 (4.1%)	55/2522 (2.2%)	1.965 (1.310–2.946)	
ARC-HBR				0.699
No	24/858 (2.8%)	39/2236 (1.7%)	1.695 (1.018–2.823)	
Yes	20/264 (7.6%)	23/573 (4.0%)	1.977 (1.085–3.602)	

DES indicates drug-eluting stent.

## Data Availability

The clinical and procedural data used to support the findings of this study are included within the article.

## Supplementary Materials

Supplementary Materials Supplementary Table 1: study definitions for major and minor ARC-HBR criteria compared with the original definitions from the ARC-HBR document; Supplementary Table 2: percent standardized differences of variables among unadjusted, propensity score matching, and IPTW-adjusted cohort; Supplementary Table 3: ischemic and bleeding outcomes according to ESC high ischemic risk criteria status from 12 to 30 months after coronary stent treatment (*n* = 9212); Supplementary Table 4: 30-month ischemic and bleeding outcomes by DAPT interruption status in the propensity score matching cohort; Supplementary Table 5: baseline characteristics in the entire population (*n* = 4,430); Supplementary Table 6: lesion and procedural characteristics in the entire population (*n* = 4,430); Supplementary Table 7: 30-month ischemic and bleeding outcomes by DAPT interruption status in the entire population (*n* = 4,430); Supplementary Table 8: primary ischemic endpoint in selected subgroups in the entire population (*n* = 4,430); Supplementary Figure 1: the differential effect of temporary or permanent DAPT interruption within the first 12 months after PCI compared with DAPT maintenance >12 months on primary ischemic and key secondary endpoints in the entire population (*n* = 4,430).
