# Supplementary material for: Thrombotic vs. Bleeding Events of Interruption of Dual Antiplatelet Therapy within 12 Months among Patients with Stent-Driven High Ischemic Risk Definition following PCI
Source: J Interv Cardiol. 2022 Jan 13;2022:3895205. doi: 10.1155/2022/3895205 (PMC8776446; doi:10.1155/2022/3895205)
Supplement: Supplementary Materials — Supplementary Table 1: study definitions for major and minor ARC-HBR criteria compared with the original definitions from the ARC-HBR document; Supplementary Table 2: percent standardized differences of variables among unadjusted, propensity score matching, and IPTW-adjusted cohort; Supplementary Table 3: ischemic and bleeding outcomes according to ESC high ischemic risk criteria status from 12 to 30 months after coronary stent treatment (n = 9212); Supplementary Table 4: 30-month ischemic and bleeding outcomes by DAPT interruption status in the propensity score matching cohort; Supplementary Table 5: baseline characteristics in the entire population (n = 4,430); Supplementary Table 6: lesion and procedural characteristics in the entire population (n = 4,430); Supplementary Table 7: 30-month ischemic and bleeding outcomes by DAPT interruption status in the entire population (n = 4,430); Supplementary Table 8: primary ischemic endpoint in selected subgroups in the entire population (n = 4,430); Supplementary Figure 1: the differential effect of temporary or permanent DAPT interruption within the first 12 months after PCI compared with DAPT maintenance >12 months on primary ischemic and key secondary endpoints in the entire population (n = 4,430). [file 3895205.f1.docx]

**SUPPLEMENTAL MATERIAL**

**Thrombotic vs Bleeding Events of Interruption of Dual Antiplatelet Therapy Within 12 Months Among Patients with Stent-Driven High Ischemic Risk Definition Following PCI**

Wang HY et al.

**Supplementary Methods**

**PCI procedures**

Unfractionated heparin was used for anticoagulation during the procedure to achieve an activated clotting time of 250 to 300 seconds. Treatment strategy for access site, revascularization treatment strategy, use of glycoprotein IIb/IIIa inhibitor, and use of intravascular imaging assessment were all carried out at the operators’ discretion. The length and diameter of the stent were not restricted.

Aspirin 300 mg and a loading dose of a P2Y12 inhibitor (clopidogrel 300 or 600 mg, or ticagrelor 180 mg) were given before intervention. After PCI, patients received DAPT with aspirin 100 mg once daily plus a maintenance dose of a P2Y12 inhibitor (clopidogrel 75 mg once daily or ticagrelor 90 mg twice daily) for at least 12 months. After the procedure, all patients were recommended to receive optimal pharmacological therapy, including statins, β-blockers, or renin-angiotensin system blockade, if indicated, following clinical guidelines [17, 16]. Patients who discontinued antiplatelet therapy as a result of clinically significant active bleeding or for other procedures were monitored carefully for cardiac events.

Supplementary Table 1: Study definitions for major and minor ARC-HBR criteria compared with the original definitions from the ARC-HBR document

| **ARC-HBR criteria** | **Study definition** | **ARC-HBR document definition^1^** |
| --- | --- | --- |
| **Major criteria** |  |  |
| Oral anticoagulation | Oral anticoagulation (with a VKA or non-VKA oral anticoagulant) at discharge of hospitalization for PCI | Anticipated use of long-term oral anticoagulation |
| Severe/end-stage CKD | eGFR <30 mL/min | No difference |
| Moderate/severe anemia | Hemoglobin <11 g/dL | No difference |
| Thrombocytopenia | Platelet count <100 x10^9^/L | No difference |
| Previous stroke | Previous ischemic stroke or ICH | Previous spontaneous ICH; traumatic ICH within the past 12 months; presence of bAVM; ischemic stroke within the past 6 months |
| Previous bleeding | Spontaneous non-intracranial major bleeding requiring hospitalization or transfusion | Spontaneous bleeding in the past 6 months or at any time, if recurrent |
|  | NA | Active malignancy within the past 12 months |
|  | NA | Non-deferrable major surgery on dual antiplatelet therapy |
|  | NA | Chronic bleeding diathesis |
|  | NA | Liver cirrhosis with portal hypertension |
|  | NA | Recent major surgery or major trauma within 30 days before PCI |
| **Minor criteria** |  |  |
| Age 75+ | Age ≥75 years | No difference |
| Moderate CKD | eGFR 30-59 mL/min | No difference |
| Mild anemia | Hemoglobin 11-12.9 g/dL for men and 11-11.9 g/dL for women | No difference |
|  | NA | Spontaneous bleeding within the past 12 months not meeting the major criterion |
|  | NA | Any ischemic stroke at any time not meeting the major criterion |
|  | NA | Long-term use of oral NSAIDs or steroids |

ARC-HBR = Academic Research Consortium for High Bleeding Risk; bAVM = brain arteriovenous malformation; CKD = chronic kidney disease; CVA = cerebrovascular accident; eGFR = estimated glomerular filtration rate; ICH = intracranial hemorrhage; NA = not available; NSAIDs = nonsteroidal anti-inflammatory drugs; PCI = percutaneous coronary intervention; VKA = vitamin K antagonist.

^1^ Definitions adapted from Urban P. et al., Circulation 2019;140:240-261.

Supplementary Table 2. Percent standardized differences of variables among unadjusted, propensity-score matching, and IPTW-adjusted cohort

|  | Standardized mean difference (%) | | |
| --- | --- | --- | --- |
|  | Unadjusted (N=3931) | Propensity-score matching (N=2240) | IPTW-adjusted (N=3931) |
| Age, years | 2.7 | 3.7 | 0.0 |
| Male | 1.4 | 0.8 | 0.1 |
| Body mass index, kg/m^2^ | 3.8 | 2.9 | 0.8 |
| Hypertension | 1.0 | 0.2 | 0.2 |
| Diabetes mellitus | 4.0 | 1.1 | 0.1 |
| Hyperlipidemia | 5.6 | 1.3 | 0.0 |
| Chronic kidney disease | 1.5 | 3.6 | 0.5 |
| Current smoker | 2.2 | 0.4 | 0.0 |
| Heart failure | 3.4 | 1.3 | 0.4 |
| Peripheral artery disease | 1.2 | 1.5 | 0.4 |
| History of myocardial infarction | 0.5 | 1.5 | 0.3 |
| Prior PCI | 0.9 | 1.3 | 0.0 |
| Prior CABG | 1.0 | 2.6 | 0.4 |
| History of stroke | 5.3 | 2.7 | 0.2 |
| History of major bleeding | 5.1 | 0.9 | 0.1 |
| LVEF, % | 0.0 | 0.8 | 0.7 |
| Acute coronary syndrome | 10.2 | 0.9 | 0.4 |
| UA/NSTEMI | 6.1 | 1.8 | 0.8 |
| STEMI | 4.5 | 1.3 | 0.7 |
| White blood cell count, 10^9^/L | 2.5 | 0.0 | 0.1 |
| Hemoglobin, g/dL | 4.3 | 0.4 | 0.1 |
| Platelet count, 10^9^/L | 2.1 | 3.7 | 0.6 |
| ARC-HBR | 7.0 | 2.0 | 0.0 |
| Multivessel CAD | 3.1 | 1.7 | 0.2 |
| Location of lesion treated |  |  |  |
| LM | 2.5 | 1.7 | 0.5 |
| LAD | 2.8 | 0.8 | 0.1 |
| LCx | 2.8 | 0.8 | 0.3 |
| RCA | 2.3 | 1.7 | 0.1 |
| Bypass graft | 0.4 | 0.0 | 0.1 |
| Target lesions morphology |  |  |  |
| Heavy calcified lesion | 0.9 | 3.0 | 0.4 |
| In-stent restenosis lesion | 0.9 | 0.9 | 0.2 |
| Bifurcation lesion | 1.4 | 2.3 | 0.0 |
| Thrombotic lesion | 2.5 | 0.4 | 0.4 |
| Chronic total occlusion | 5.1 | 1.9 | 0.0 |
| Type B2 or C lesion | 5.2 | 1.5 | 0.0 |
| SYNTAX score | 1.7 | 0.8 | 0.6 |
| Total lesion length, mm | 5.0 | 1.9 | 0.3 |
| Number of vessels treated | 2.0 | 1.2 | 0.2 |
| Number of lesions treated | 0.0 | 0.7 | 0.1 |
| Number of stents implanted | 5.8 | 1.2 | 0.9 |
| Total stent length, mm | 3.5 | 1.2 | 0.5 |
| Mean stent diameter, mm | 1.6 | 2.2 | 0.8 |
| Vascular access site | 2.6 | 0.0 | 0.1 |
| Radial approach |  |  |  |
| Femoral approach |  |  |  |
| Use of intravascular ultrasound | 1.8 | 1.6 | 0.1 |
| Use of glycoprotein IIb/IIIa inhibitors | 3.5 | 1.6 | 0.1 |
| Drug-eluting stent type | 0.2 | 1.8 | 0.2 |
| First-generation DES |  |  |  |
| Second-generation DES |  |  |  |

CAD indicates coronary artery disease; ACS indicates acute coronary syndrome; ARC-HBR, Academic Research Consortium-High Bleeding Risk; CABG, coronary artery bypass grafting; DAPT, dual antiplatelet therapy; DES, drug-eluting stent; LVEF, left ventricular ejection fraction; LM, left main coronary artery; LAD, left anterior descending coronary artery; LCx, left circumﬂex coronary artery; NSTEMI, non-ST-segment elevation myocardial infarction; RCA, right coronary artery; and SYNTAX, Synergy Between PCI With Taxus and Cardiac Surgery; STEMI, ST-segment elevation myocardial infarction; and UA, unstable angina.

Supplementary Table 3: Ischemic and bleeding outcomes according to ESC high ischemic risk criteria status from 12 to 30 months after coronary stent treatment (n=9212)

|  | High ischemic risk (n=3,931) | No high ischemic risk  (n=5,281) | Unadjusted  HR (95% CI) | P Value | Adjusted  HR (95% CI)* | P Value |
| --- | --- | --- | --- | --- | --- | --- |
| Major adverse cardiac and cerebrovascular events ^a^ | 106 (2.7) | 86 (1.6) | 1.660 (1.248-2.206) | <0.001 | 1.533 (1.150-2.044) | 0.004 |
| All-cause death | 41 (1.0) | 33 (0.6) | 1.675 (1.059-2.648) | 0.028 | 1.526 (0.959-2.427) | 0.075 |
| Cardiac death | 25 (0.6) | 14 (0.3) | 2.405 (1.250-4.626) | 0.009 | 2.122 (1.092-4.122) | 0.026 |
| Myocardial infarction | 30 (0.8) | 25 (0.5) | 1.622 (0.954-2.757) | 0.074 | 1.455 (0.850-2.489) | 0.171 |
| Stent thrombosis (definite/probable) | 17 (0.4) | 11 (0.2) | 2.092 (0.980-4.465) | 0.057 | 1.949 (0.905-4.197) | 0.088 |
| Stroke | 57 (1.5) | 33 (0.6) | 2.315 (1.506-3.556) | <0.001 | 2.138 (1.385-3.299) | 0.001 |
| Clinically relevant bleeding ^b^ | 43 (1.1) | 63 (1.2) | 0.909 (0.616-1.341) | 0.630 | 0.908 (0.613-1.346) | 0.632 |

Values are number of events (Kaplan-Meier estimated event rates), unless otherwise indicated. *Adjusted variables included age, sex, body mass index, current smoking, hypertension, hyperlipidemia, peripheral artery disease, prior myocardial infarction, prior PCI, prior coronary artery bypass grafting surgery, and acute coronary syndrome presentation.

^a^ Major adverse cardiac and cerebrovascular events included the composite of all-cause death, myocardial infarction, or stroke;

^b^ Clinically relevant bleeding was defined as BARC type 2, 3, or 5 bleeding;

Supplementary Table 4: 30-month ischemic and bleeding outcomes by DAPT interruption status in the propensity score-matching cohort

|  | DAPT interruption ≤12-month (n=1120) | DAPT maintenance >12-month (n=1120) |
| --- | --- | --- |
| Major adverse cardiac and cerebrovascular events | 44 (3.9%) | 23 (2.1%) |
| All-cause death | 20 (1.8%) | 8 (0.7%) |
| Cardiac death | 16 (1.4%) | 3 (0.3%) |
| Myocardial infarction | 15 (1.3%) | 5 (0.4%) |
| Stent thrombosis | 9 (0.8%) | 3 (0.3%) |
| Stroke | 19 (1.7%) | 15 (1.3%) |
| Clinically relevant bleeding | 11 (1.0%) | 15 (1.3%) |
| Net adverse clinical events | 55 (4.9%) | 36 (3.2%) |

Values are number of events (%) unless otherwise indicated. BARC indicates Bleeding Academic Research Consortium; DAPT, dual antiplatelet therapy; MACCE, major adverse cardiac and cerebrovascular events; MI, myocardial infarction; and ST, stent thrombosis.

Supplementary Table 5: Baseline characteristics in the entire population (n=4,430)

|  | Interruption of DAPT  within 12 months after PCI | |  |
| --- | --- | --- | --- |
|  | No (n=3160) | Yes (n=1270) | P value |
| Age, years | 59.03 ± 10.15 | 59.66 ± 10.56 | 0.064 |
| Male | 2429 (76.9) | 967 (76.1) | 0.606 |
| Hyperlipidemia | 2194 (69.4) | 848 (66.8) | 0.084 |
| Hypertension | 2159 (68.3) | 868 (68.3) | 0.988 |
| Diabetes mellitus | 1472 (46.6) | 601 (47.3) | 0.655 |
| Chronic kidney disease* | 285 (9.0) | 119 (9.4) | 0.714 |
| Current smoker | 1800 (57.0) | 709 (55.8) | 0.491 |
| Heart failure | 77 (2.4) | 33 (2.6) | 0.754 |
| Peripheral artery disease | 100 (3.2) | 47 (3.7) | 0.368 |
| History of myocardial infarction | 678 (21.5) | 262 (20.6) | 0.543 |
| Prior PCI | 706 (22.3) | 301 (23.7) | 0.329 |
| Prior CABG | 157 (5.0) | 62 (4.9) | 0.904 |
| History of stroke | 376 (11.9) | 177 (13.9) | 0.063 |
| History of major bleeding^†^ | 20 (0.6) | 15 (1.2) | 0.062 |
| Body mass index, kg/m^2^ | 26.07 ± 3.12 | 25.96 ± 3.29 | 0.294 |
| LVEF, % | 62.39 ± 7.60 | 62.33 ± 7.40 | 0.804 |
| Clinical presentation |  |  | 0.006 |
| Stable coronary artery disease | 1400 (44.3) | 505 (39.8) |  |
| Acute coronary syndrome | 1760 (55.7) | 765 (60.2) |  |
| UA/NSTEMI | 1388 (43.9) | 592 (46.6) | 0.103 |
| STEMI | 372 (11.8) | 173 (13.6) | 0.090 |
| White blood cell count, 10^9^/L | 6.80 ± 1.63 | 6.83 ± 1.64 | 0.683 |
| Hemoglobin, g/dL | 14.23 ± 1.58 | 14.18 ± 1.55 | 0.280 |
| Platelet count, 10^9^/L | 204.29 ± 54.16 | 205.47 ± 54.15 | 0.514 |
| ARC-HBR | 612 (19.4) | 292 (23.0) | 0.007 |
| Discharge medication |  |  |  |
| Aspirin | 3125 (98.9) | 1258 (99.1) | 0.633 |
| Clopidogrel | 3113 (98.5) | 1255 (98.8) | 0.433 |
| Ticagrelor | 11 (0.3) | 11 (0.9) | 0.027 |
| β-blocker | 2904 (91.9) | 1167 (91.9) | 0.992 |
| ACEI/ARB | 1926 (60.9) | 778 (61.3) | 0.848 |
| CCB | 1586 (50.2) | 620 (48.8) | 0.409 |
| Statin | 3037 (96.1) | 1208 (95.1) | 0.137 |

Values are mean ± SD for continuous variables, and n (%) for categorical variables. ACS indicates acute coronary syndrome; ACEI, angiotensin-converting enzyme inhibitors; ARB, angiotensin II receptor antagonists; ARC-HBR, Academic Research Consortium-High Bleeding Risk; CCB, Calcium channel blockers; CABG, coronary artery bypass grafting; DAPT, dual antiplatelet therapy; LVEF, left ventricular ejection fraction; NSTEMI, non-ST-segment elevation myocardial infarction; PCI, percutaneous coronary intervention; STEMI, ST-segment elevation myocardial infarction; and UA, unstable angina. *Chronic kidney disease was defined as an estimated glomerular filtration rate of less than 60 mL/min/1.73 m^2^ of body surface area. ^†^Defined as spontaneous (nonintracranial) bleeding requiring hospitalization or transfusion.

Supplementary Table 6: Lesion and procedural characteristics in the entire population (n=4,430)

|  | Interruption of DAPT  within 12 months after PCI | |  |
| --- | --- | --- | --- |
|  | No (n=3160) | Yes (n=1270) | P value |
| **Lesion characteristics** |  |  |  |
| Multivessel CAD | 2862 (90.6) | 1134 (89.3) | 0.196 |
| Location of lesion treated |  |  |  |
| LM | 152 (4.8) | 65 (5.1) | 0.668 |
| LAD | 2669 (84.5) | 1091 (85.9) | 0.225 |
| LCx | 949 (30.0) | 394 (31.0) | 0.516 |
| RCA | 1071 (33.9) | 410 (32.3) | 0.305 |
| Bypass graft | 7 (0.2) | 4 (0.3) | 0.572 |
| Target lesions morphology |  |  |  |
| Heavy calcified lesion | 165 (5.2) | 64 (5.0) | 0.804 |
| In-stent restenosis lesion | 146 (4.6) | 55 (4.3) | 0.675 |
| Bifurcation lesion | 624 (19.7) | 262 (20.6) | 0.506 |
| Bifurcation with two stents implanted | 316 (10.0) | 112 (8.8) | 0.229 |
| Thrombotic lesion | 135 (4.3) | 59 (4.6) | 0.583 |
| Chronic total occlusion | 612 (19.4) | 224 (17.6) | 0.183 |
| Type B2 or C lesion | 2834 (89.7) | 1153 (90.8) | 0.268 |
| SYNTAX score | 14.71 ± 0.52 | 2.91 ± 0.54 | 0.642 |
| Total lesion length, mm | 57.69 ± 30.93 | 55.84 ± 28.18 | 0.065 |
| **Procedural characteristics** |  |  |  |
| Number of vessels treated | 1.49 ± 0.59 | 1.50 ± 0.59 | 0.641 |
| Number of lesions treated | 1.75 ± 0.82 | 1.75 ± 0.81 | 0.886 |
| 1 | 1403 (44.4) | 573 (45.1) | 0.663 |
| 2 | 1241 (39.3) | 480 (37.8) | 0.362 |
| ≥3 | 516 (16.3) | 217 (17.1) | 0.557 |
| Number of stents implanted | 2.66 ± 1.15 | 2.60 ± 1.13 | 0.123 |
| 1 | 479 (15.2) | 210 (16.5) | 0.253 |
| 2 | 974 (30.8) | 381 (30.0) | 0.591 |
| ≥3 | 1707 (54.0) | 678 (53.4) | 0.702 |
| Total stent length, mm |  |  | 0.237 |
| Total stent length>60 mm | 1458 (46.1) | 594 (46.8) | 0.703 |
| Mean stent diameter, mm | 2.91 ± 0.52 | 2.91 ± 0.54 | 0.945 |
| Vascular access site | 61.09 ± 29.12 | 59.96 ± 27.92 | 0.533 |
| Radial approach | 2856 (90.4) | 1140 (89.8) |  |
| Femoral approach | 304 (9.6) | 130 (10.2) |  |
| Use of intravascular ultrasound | 234 (7.4) | 104 (8.2) | 0.374 |
| Use of glycoprotein IIb/IIIa inhibitors | 623 (19.7) | 262 (20.6) | 0.491 |
| Drug-eluting stent type |  |  | 0.901 |
| First-generation DES | 322 (10.2) | 131 (10.3) |  |
| Second-generation DES | 2838 (89.8) | 1139 (89.7) |  |

Values are mean ± SD for continuous variables, and n (%) for categorical variables. CAD indicates coronary artery disease; DES, drug-eluting stent; LM, left main coronary artery; LAD, left anterior descending coronary artery; LCx, left circumﬂex coronary artery; RCA, right coronary artery; and SYNTAX, Synergy Between PCI With Taxus and Cardiac Surgery.

Supplementary Table 7**:** 30-month ischemic and bleeding outcomes by DAPT interruption status in the entire population (n=4,430)

|  | DAPT interruption ≤12-month (n=1270) | DAPT maintenance  >12-month (n=3160) | Multivariable-Adjusted* | | Propensity-Score Matching | | IPTW-Adjusted | | Unadjusted | |
| --- | --- | --- | --- | --- | --- | --- | --- | --- | --- | --- |
|  |  |  | HR (95% CI) | P value | HR (95% CI) | P value | HR (95% CI) | P value | HR (95% CI) | P value |
| Major adverse cardiac and cerebrovascular events ^a^ | 94 (7.4) | 158 (5.0) | 1.501 (1.161-1.941) | 0.002 | 1.511 (1.096-2.084) | 0.012 | 1.480 (1.134-1.930) | 0.004 | 1.547 (1.198-1.997) | 0.001 |
| All-cause death | 42 (3.3) | 26 (0.8) | 4.133 (2.525-6.767) | <0.001 | 3.167 (1.690-5.937) | <0.001 | 3.990 (2.182-7.294) | <0.001 | 4.253 (2.606-6.940) | <0.001 |
| Cardiac death | 31 (2.4) | 11 (0.3) | 7.173 (3.588-14.399) | <0.001 | 4.413 (1.931-10.085) | <0.001 | 6.999 (2.830-17.308) | <0.001 | 7.416 (3.725-14.766) | <0.001 |
| Myocardial infarction | 47 (3.7) | 76 (2.4) | 1.559 (1.081-2.247) | 0.017 | 1.886 (1.159-3.070) | 0.011 | 1.557 (1.062-2.281) | 0.023 | 1.574 (1.095-2.267) | 0.014 |
| Stent thrombosis (definite/probable) | 25 (2.0) | 23 (0.7) | 2.807 (1.590-4.958) | <0.001 | 2.777 (1.290-5.979) | 0.009 | 2.912 (1.505-5.633) | 0.002 | 2.832 (1.607-4.993) | <0.001 |
| Stroke | 33 (2.6) | 59 (1.9) | 1.369 (0.890-2.106) | 0.153 | 1.364 (0.808-2.304) | 0.245 | 1.378 (0.890-2.135) | 0.151 | 1.485 (0.969-2.275) | 0.069 |
| Clinically relevant bleeding ^b^ | 44 (3.5) | 69 (2.2) | 1.680 (1.144-2.466) | 0.008 | 1.607 (1.004-2.572) | 0.048 | 1.642 (1.104-2.444) | 0.014 | 1.746 (1.193-2.556) | 0.004 |
| Net adverse clinical events ^c^ | 130 (10.2) | 221 (7.0) | 1.516 (1.219-1.887) | <0.001 | 1.527 (1.163-2.004) | 0.002 | 1.493 (1.192-1.868) | <0.001 | 1.561 (1.256-1.939) | <0.001 |

Values are number of events (Kaplan-Meier estimated event rates), unless otherwise indicated. *Adjusted variables included age, sex, body mass index, current smoking, hypertension, diabetes mellitus, left ventricular ejection fraction, peripheral artery disease, prior coronary artery bypass grafting, prior myocardial infarction, prior PCI, prior major bleeding, acute coronary syndrome presentation, transradial approach, use of intravascular ultrasound, drug-eluting stent type, and total stent length.

^a^ Major adverse cardiac and cerebrovascular events included the composite of all-cause mortality, myocardial infarction, or stroke;

^b^ Clinically relevant bleeding was defined as BARC type 2, 3, or 5 bleeding;

^c^ Net adverse clinical events included the composite of all-cause mortality, myocardial infarction, stroke, or clinically relevant bleeding.

CI, confidence interval; CABG, coronary artery bypass grafting; DAPT, dual antiplatelet therapy; HR, hazard ratio; PCI, percutaneous coronary intervention.

Supplementary Table 8: Primary ischemic endpoint in selected subgroups in the entire population (n=4,430)

|  | DAPT interruption ≤12-month (n=1270) | DAPT maintenance  >12-month (n=3160) | HR (95% CI) | P for interaction |
| --- | --- | --- | --- | --- |
| Age |  |  |  | 0.093 |
| <65 years | 40/847 (4.7%) | 91/2226 (4.1%) | 1.197 (0.825-1.737) |  |
| ≥65 years | 54/423 (12.8%) | 67/934 (7.2%) | 1.876 (1.310-2.685) |  |
| Sex |  |  |  | 0.431 |
| Female | 27/303 (8.9%) | 37/731 (5.1%) | 1.804 (1.098-2.963) |  |
| Male | 67/967 (6.9%) | 121/2429 (5.0%) | 1.461 (1.084-1.970) |  |
| Diabetes mellitus |  |  |  | 0.697 |
| No | 56/669 (8.4%) | 91/1688 (5.4%) | 1.600 (1.146-2.233) |  |
| Yes | 38/601 (6.3%) | 67/1472 (4.6%) | 1.464 (0.983-2.182) |  |
| Chronic kidney disease |  |  |  | 0.454 |
| No | 78/1151 (6.8%) | 126/2875 (4.4%) | 1.623 (1.223-2.153) |  |
| Yes | 16/119 (13.4%) | 32/285 (11.2%) | 1.249 (0.685-2.276) |  |
| Smoking |  |  |  | 0.275 |
| No | 48/561 (8.6%) | 68/1360 (5.0%) | 1.783 (1.232-2.580) |  |
| Yes | 46/709 (6.5%) | 90/1800 (5.0%) | 1.359 (0.952-1.939) |  |
| Acute coronary syndrome |  |  |  | 0.714 |
| No | 39/505 (7.7%) | 70/1400 (5.0%) | 1.616 (1.092-2.392) |  |
| Yes | 55/765 (7.2%) | 88/1760 (5.0%) | 1.481 (1.056-2.076) |  |
| Previous MI |  |  |  | 0.185 |
| No | 76/1008 (7.5%) | 115/2482 (4.6%) | 1.696 (1.269-2.267) |  |
| Yes | 18/262 (6.9%) | 43/678 (6.3%) | 1.199 (0.644-1.944) |  |
| Multivessel disease |  |  |  | 0.984 |
| No | 8/136 (5.9%) | 12/298 (4.0%) | 1.539 (0.628-3.768) |  |
| Yes | 86/1134 (7.6%) | 146/2862 (5.1%) | 1.543 (1.182-2.015) |  |
| Generation of DES |  |  |  | 0.861 |
| First-generation DES | 9/131 (6.9%) | 16/322 (5.0%) | 1.486 (0.654-3.377) |  |
| Second-generation DES | 85/1139 (7.5%) | 142/2838 (5.0%) | 1.556 (1.189-2.036) |  |
| ARC-HBR |  |  |  | 0.295 |
| No | 57/978 (5.8%) | 113/2548 (4.4%) | 1.364 (0.992-1.876) |  |
| Yes | 37/292 (12.7%) | 45/612 (7.4%) | 1.822 (1.179-2.815) |  |

DES indicates drug-eluting stent.

Supplementary Figure 1: The differential effect of temporary or permanent DAPT interruption within the first 12 months after PCI compared with DAPT maintenance >12-month on primary ischemic and key secondary endpoints in the entire population (n=4,430)


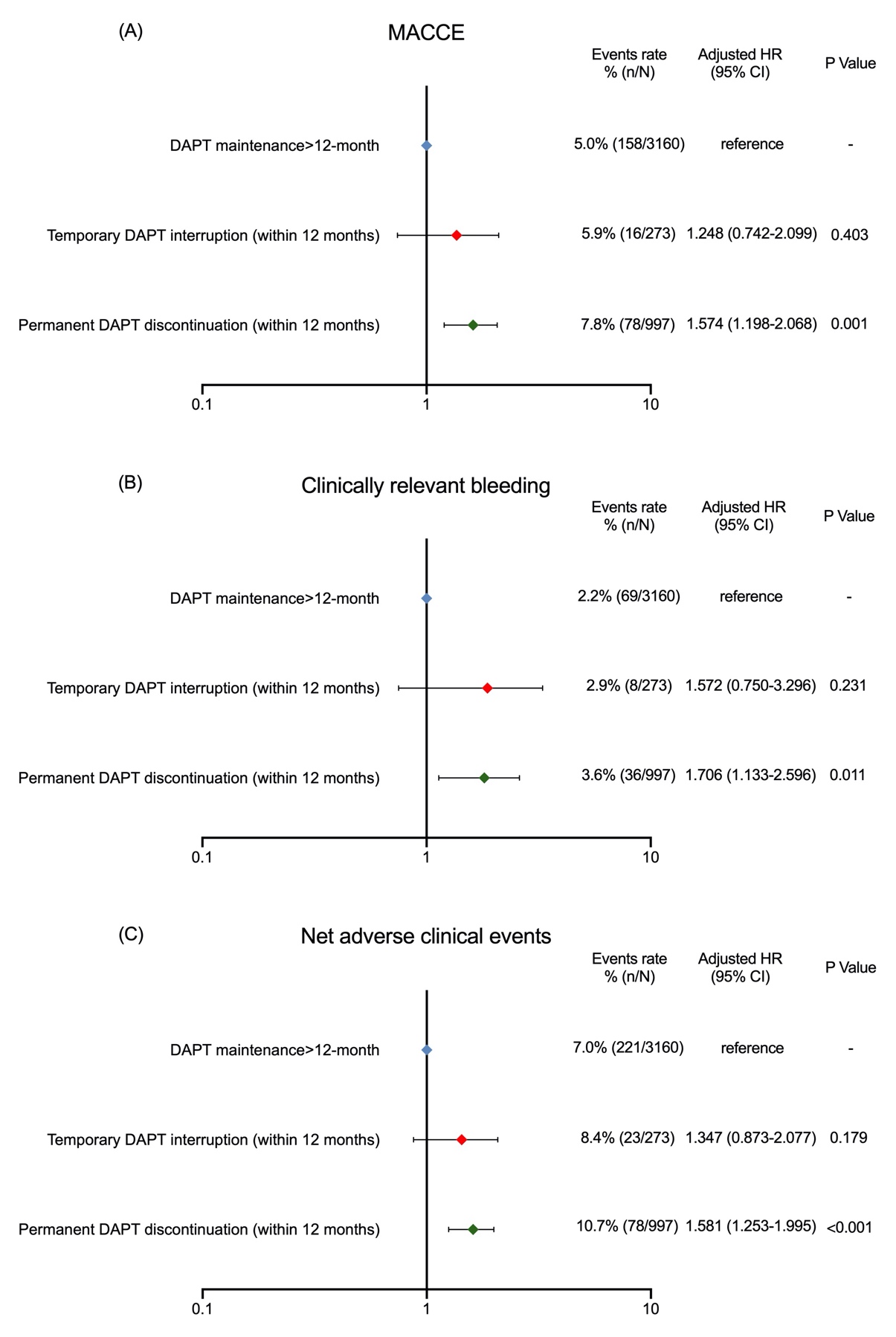


MACCE = Major adverse cardiac and cerebrovascular events
